# Review of the *Fannia postica*-group Chillcott, 1961 of the genus *Fannia* Robineau-Desvoidy, 1830, with description of two new species from the Palearctic and Oriental regions (Diptera, Fanniidae)

**DOI:** 10.3897/zookeys.598.7983

**Published:** 2016-06-14

**Authors:** Ming-fu Wang, Wei Li, Wei-bing Zhu, Dong Zhang

**Affiliations:** 1 Institute of Entomology, Shenyang Normal University, Shenyang, 110034, P.R. China; 2 College of Biological Sciences and Biotechnology, Beijing Forestry University, Beijing, 100083, P.R. China; 3 Shanghai Entomological Museum, Chinese Academy of Sciences, Shanghai, 200032, P.R. China; 4 School of Nature Conservation, Beijing Forestry University, Beijing 100083, P.R. China

**Keywords:** Species transference, Fannia serena-group, identification key, new Chinese species

## Abstract

A total of 17 species of the *Fannia postica*-group Chillcott, 1961 from the Palearctic and Oriental regions are reviewed herein, 2 of which are described from China as new: *Fannia ningxiaensis* Wang & Zhang, **sp. n.** and *Fannia subaethiops* Wang & Zhu, **sp. n.**. *Fannia labidocerca* Feng & Xue, 2006, originally placed in *Fannia serena*-group Chillcott, 1961, is moved to the *postica*-group and re-described. An identification key to the males of known species from these regions is provided.

## Introduction

The *Fannia postica*-group was established in the genus *Fannia* Robineau-Desvoidy, 1830 by Chillcott (1961). Species of this group are classified under two subgroups, the *Fannia postica*-subgroup and the *Fannia spathiophora*-subgroup (Chillcott 1961). The group is currently composed of approximately 25 known species worldwide (including the species added in this paper), most of which are distributed in the Holarctic Region, with a minority of species in the Oriental Region (Wang et al. 2011). They are: *Fannia postica*-subgroup: *Fannia brevicauda* Chillcott, *Fannia discoculea* Xue, *Fannia enigmata* Chillcott, *Fannia flavibasis* (Stein), *Fannia labidocerca* Feng & Xue, *Fannia multisetosa* Chillcott, *Fannia postica* (Stein), *Fannia ringdahlana* Collin, *Fannia sequoiae* Chillcott; *Fannia spathiophora*-subgroup: *Fannia aethiops* Malloch, *Fannia ardua* Nishida, *Fannia bigelowi* Chillcott, *Fannia brooksi* Chillcott, *Fannia coculea* Nishida, *Fannia gotlandica* Ringdahl, *Fannia ningxiaensis* Wang & Zhang, sp. n., *Fannia nudifemorata* Wang & Zhang, *Fannia scyphocerca* Chillcott, *Fannia slovaca* Gregor & Rozkošný, *Fannia spathiophora* Malloch, *Fannia stigi* Rognes, *Fannia subaethiops* Wang & Zhu, sp. n., *Fannia tundrarum* Chillcott, *Fannia umbratica* Collin, *Fannia umbrosa* (Stein).

At the end of the nineteenth century, Stein (1895) described the Palearctic and Oriental species, *Fannia postica* (Stein). Since the beginning of the twentieth century, a number of papers and monographs studying the European species of *Fannia postica*-group have been published (Ringdahl 1926, Collin 1939, Hennig 1955, D’Assis-Fonseca 1968, Rognes 1982, Gregor and Rozkošný 2005). Pont (1986) reviewed the Palearctic fanniids, including species of the *Fannia postica*-group. Rozkošný et al. (1997) treated species of the Family Fanniidae from Europe and added nine species to the *Fannia postica*-group.

Asian species of the *Fannia postica*-group were mainly reported on by Nishida (1975, 1976), Pont (1977), Fan (1992), Xue and Wang (1998), Xue et al. (2001), Wang and Xue (2002) and Wu and Wang (2002). Wang et al. (2011) listed 22 known species of this group worldwide, recorded nine species of the *postica*-group from China and, based on the definition of the group by Chillcott (1961), summarized features of the male habitus and terminalia that distinguish it from other Fanniidae.

The biological characteristics of these species have never been fully studied. The study of specimens in our entomological collections has revealed that the majority of species in the group occur in wooded or shrubby habitats. According to Rozkošný et al. (1997), the immature stages of some species, such as *Fannia umbrosa* (Stein), live in birds’ nests or in sap flowing out of rotholes in trees. The larvae of *Fannia postica* (Stein) and *Fannia umbrosa* (Stein) feed on humic substances, whereas the larvae of *Fannia postica* (Stein) also develop in carrion (Rozkošný et al. 1997).

In this paper, we review 17 known Palearctic and Oriental species belonging to the *Fannia postica*-group. Based on an extensive literature search and study of dry specimens, a key to the identification of males of known species from these regions is given, and two new species from China are described. One species, *Fannia labidocerca* Feng & Xue, 2006, is transferred from the *Fannia serena*-group to the *Fannia postica*-group and re-described. Illustrations of the male terminalia are included.

## Material and methods

The morphological terminology used in this paper follows McAlpine (1981), except for the term “postpedicel”, which follows Stuckenberg (1999). Absolute measurements in millimeters (mm) are given for body length. The specimens studied for this paper are deposited in the Institute of Entomology, Shenyang Normal University, Shenyang, China (IESNU) and the Shanghai Entomological Museum, Chinese Academy of Science, Shanghai, China (SHEM). Figure of *Fannia labidocerca* Feng & Xue, 2006 is from Feng and Xue (2006). Methods for the preparation of terminalia and illustrations follow Zhang et al. (2013).

The following abbreviations are used for characters throughout the text: *acr* = acrostichal seta(e), *ad* = anterodorsal seta(e), *av* = anteroventral seta(e), *d* = dorsal seta(e), *dc* = dorsocentral seta(e), *ia* = intra-alar seta(e), *p* = posterior seta(e), *pd* = posterodorsal seta(e), *pra* = prealar seta(e), and *pv* = posteroventral seta(e).

## Taxonomic accounts

### Genus Fannia

Taxon classificationAnimaliaDipteraFanniidae

Robineau-Desvoidy, 1830

Fannia postica -group: Chillcott 1961: 101, 222; Rozkošný et al. 1997: 48; Wang et al. 2011: 3.
Fannia For a diagnosis of the group see Wang et al. (2011).

#### Key to males of the known Palearctic and Oriental species in the *Fannia postica*-group

**Table:** 

1	Hind femur with at least 2 *av* in distal half (*Fannia postica*-subgroup)	**2**
–	Hind femur with only 1 *av* in distal half (*Fannia spathiophora*-subgroup)	**5**
2	*Pra* 1; hind coxa with setulae on posterior surface	***Fannia discoculea* Xue**
–	*Pra* 2; hind coxa bare on posterior surface	**3**
3	Mid first tarsomere without a basal tooth-like spine on ventral surface; hind femur with 4 to 6 *av* in distal half; calypters yellow	***Fannia postica* (Stein)**
–	Mid first tarsomere with a basal tooth-like spine on ventral surface; hind femur with only 2 *av* in distal half; calypters blackish	**4**
4	Hind femur without distinct *pv*, and with 3 to 5 *av* in distal half	***Fannia labidocerca* Feng & Xue**
–	Hind femur with 7 or 8 *pv* in distal half, and with 2 *av* in distal half	***Fannia ringdahlana* Collin**
5	Hind coxa with setulae on posterior surface; *pra* 2 (rarely 3); frontal setae 7 to 9; mid first tarsomere with a basal tooth-like spine on ventral surface	***Fannia coculea* Nishida**
–	Hind coxa bare on posterior surface	**6**
6	Fore tibia with 7 to 9 slender *pv*	***Fannia spathiophora* Malloch**
–	Fore tibia without slender *pv*	**7**
7	Hind femur without distinct *pv*; haltere brown	***Fannia nudifemorata* Wang & Zhang**
–	Hind femur with *pv*	**8**
8	Hind femur with 3 to 5 *pv* in distal half	**9**
–	Hind femur with 7 to 14 *pv* in distal half	**13**
9	Abdomen at least yellowish in basal part	***Fannia gotlandica* Ringdahl**
–	Abdomen entirely black	**10**
10	Mid first tarsomere with a stout basal tooth-like spine on ventral surface	***Fannia stigi* Rognes**
–	Mid first tarsomere with a weak basal tooth-like spine on ventral surface	**11**
11	Syntergite 1+2 and tergites 3–4 each with a dark median stripe	***Fannia aethiops* Malloch**
–	Syntergite 1+2 and tergites 3–4 each with an inverted T-shaped dark mark	**12**
12	Frons, at its narrowest point, about as wide as anterior ocellus; *pra* short and weak, the anterior one about 1/2 as long as the length of posterior notopleural seta	***Fannia ardua* Nishida**
–	Frons, at its narrowest point, slightly wider than the distance between outer margins of posterior ocelli; *pra* slightly stout, the anterior one about 2/3 as long as the length of posterior notopleural seta	***Fannia subaethiops* Wang & Zhu, sp. n.**
13	Postocular setae in 2 rows	**14**
–	Postocular setae in one row	**15**
14	*Acr* mainly triserial; mid tibia strongly flattened and with a posteroventral ridge	***Fannia bigelowi* Chillcott**
–	*Acr* mainly biserial; mid tibia not strongly flattened and without a posteroventral ridge	***Fannia ningxiaensis* Wang & Zhang, sp. n.**
15	Scutum entirely black; bacilliform process long and only bent ventrally	***Fannia umbratica* Collin**
–	Scutum with thin grayish pollinosity; bacilliform process long or short, twisted	**16**
16	Hind femur with 10 to 15 stout *pv*; bacilliform process short	***Fannia umbrosa* (Stein)**
–	Hind femur with 5 stout *pv*; bacilliform process long	***Fannia slovaca* Gregor & Rozkošný**

### Catalog of known Palearctic and Oriental species in the *Fannia postica*-group, with redescription of one species and description of two new species

#### Fannia aethiops

Taxon classificationAnimaliaDipteraFanniidae

Malloch, 1913

Fannia aethiops Malloch, 1913: 628.
Fannia aethiops : Pont 1986: 44; Rozkošný et al. 1997: 23; Xue and Wang 1998: 813; Wang and Xue 2002: 55; Wu and Wang 2002: 563; Wang et al. 2004: 34; Wang et al. 2006: 555.

##### Material examined.

China: Jilin: 1 male, Mt. Changbai, 42.33°N, 127.27°E, 22.VI.1980, Coll. Z.Y. Ma (IESNU). Shanxi: 1 male, Ningwu, Mt. Luya, 38.73°N, 111.93°E, 12.VI.1987, Coll. M.F. Wang (IESNU).

##### Distribution.

Nearctic: throughout Canada, USA (Alaska, North Carolina, south to California); Palearctic: China (Jilin, Neimenggu, Shanxi), Sweden.

#### Fannia ardua

Taxon classificationAnimaliaDipteraFanniidae

Nishida, 1976

Fannia ardua Nishida, 1976: 135.
Fannia ardua : Pont 1986: 44; Wang and Xue 2002: 55; Wang et al. 2006: 555.

##### Material examined.

China: Jilin: 1 male, Mt. Changbai, 42.33°N, 127.27°E, 10.VII.1998 (IESNU).

##### Distribution.

Palearctic: China (Jilin), Japan.

#### Fannia bigelowi

Taxon classificationAnimaliaDipteraFanniidae

Chillcott, 1961

Fannia bigelowi Chillcott, 1961: 115.
Fannia bigelowi : Pont 1986: 45.

##### Distribution.

Nearctic: Canada, USA (Alaska); Palearctic: Norway.

#### Fannia coculea

Taxon classificationAnimaliaDipteraFanniidae

Nishida, 1975

Fannia coculea Nishida, 1975: 368.
Fannia cocula : Pont 1977: 448; Xue and Wang 1998: 815; Wang and Xue 2002: 56.

##### Distribution.

Oriental: China (Taiwan).

#### Fannia discoculea

Taxon classificationAnimaliaDipteraFanniidae

Xue, 1998

Fannia discoculea Xue, 1998: 815.
Fannia discoculea : Wang and Xue 2002: 56.

##### Type specimens examined.

Holotype male: China, Xinjiang, Jakesi, 43.82°N, 81.12°E, 6.VIII.1957, Coll. G. Wang (IESNU).

##### Distribution.

Palearctic: China (Xinjiang).

#### Fannia gotlandica

Taxon classificationAnimaliaDipteraFanniidae

Ringdahl, 1926

Fannia gotlandica Ringdahl, 1926: 106.
Fannia gotlandica : Pont 1986: 48; Rozkošný et al. 1997: 39.

##### Distribution.

Palearctic: throughout Europe.

#### Fannia labidocerca

Taxon classificationAnimaliaDipteraFanniidae

Feng & Xue, 2006

Fig. 1

Fannia labidocerca Feng & Xue, 2006: 217.

##### Redescription.

**Figure 1.**
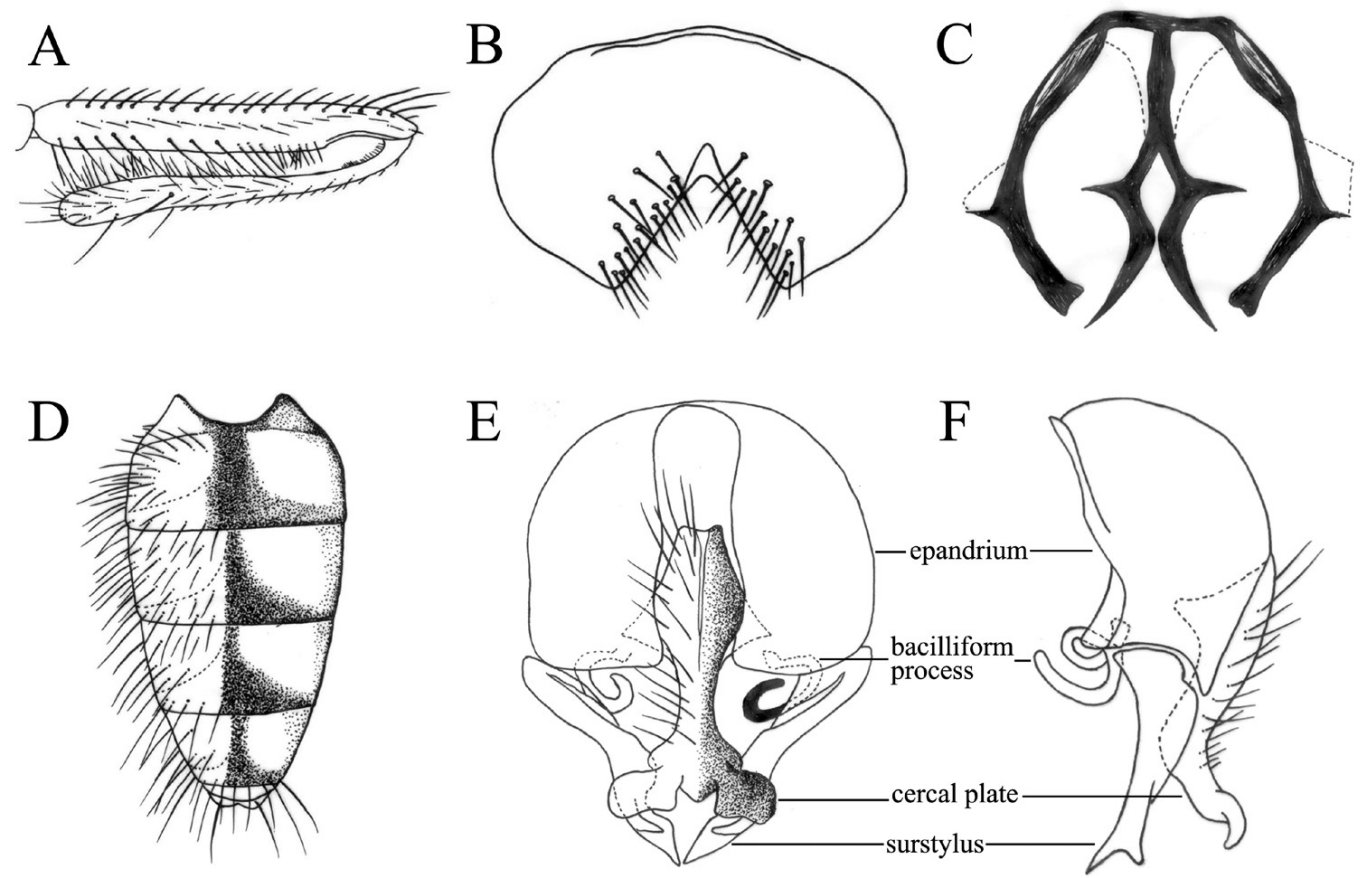
*Fannia labidocerca* Feng & Xue, 2006, male, holotype: (A−F in figure 1 without scale are all from Feng & Xue 2006, specimen from Sichuan, deposited in IESNU). **A** Mid leg, anterior view **B** Sternite 5, ventral view **C** Sternite 9, ventral view. **D.** Abdomen, dorsal view **E** Terminalia, ventral view **F** Terminalia, lateral view.

MALE. Body length 4.8 mm. Eye with short and distant hairs or bare; postocular setae in 2 rows, those of the anterior row sparse and long, curved anteriorly, those of the posterior row short; fronto-orbital plate and parafacial with silvery-white pollinosity; frons at narrowest point slightly wider than the distance between outer margins of posterior ocelli; frontal vitta black, at narrowest point about as wide as fronto-orbital plate; frontal setae 12, stout, situated on the lower 4/5 of frons, orbital setae absent; parafacial bare, at middle about 1/3 as wide as the width of postpedicel; antenna black, postpedicel about 2.0 to 2.5x as long as wide, arista ciliated, the longest individual hairs shorter than aristal base; epistoma not projecting beyond vibrissal angle, vibrissal angle behind frontal angle in profile; genal height about 1/14 of eye height; prementum shining, about 3.0x as long as wide; palpus dark brown, claviform, slightly shorter than prementum. Thorax ground-color black, notum with dark brown pollinosity; presutural *acr* biserial, long, one pair of them slightly stout, only prescutellar pairs stout, *dc* 2+3, *ia* 0+2, *pra* 2, about 2/5 of length of posterior notopleural seta; notopleuron bare; basisternum, proepisternum, anepimeron, meron and katepimeron bare; katepisternal setae 1+1, katepisternum without a ventral spine; spiracles brown; calypters mostly brown or brownish, brownish on the outer margin, the lower one small and tongue-like, about 1/2 as long as the upper one. Wing brownish; veins and wing-base yellow; basicosta brownish-yellow; costal spine inconspicuous; node of Rs bare on ventral and dorsal surfaces; vein M_1+2_ straight, parallel to vein R_4+5_ distally; crossveins not clouded; haltere brown in basal part, yellowish at middle and dark brown in distal part. Legs entirely black, sometimes dark brown or brown; fore tibia without *p*; mid coxa without any hook-like spine or spine-like seta; mid femur concave on ventral surface in apical part, becoming swollen from distal 1/3 towards basal part, with a row of *av*, stout in basal part, becoming shorter and denser in distal 1/4, with a cluster of spine-like setae in distal 1/3, a complete row of *ad*, slightly short (Fig. 1A), with a complete row of slender *p*, slightly situated on the posteroventral surface, with a row of *pv* in basal 4/5, and with a row of setulae at middle towards distal 1/4; mid tibia slightly swollen towards apex, in distal half with one *ad*, one preapical *d*, one *pd*, and with numerous slender setulae on ventral surface, most of the setulae longer than mid tibial width in distal part (Fig. 1A); mid first tarsomere with a basal tooth-like spine on ventral surface; hind coxa bare on posterior surface; hind femur with 3 stout *av* in distal 1/3, without *pv*; hind tibia with one *av*, one *ad* and one median *d*. Abdomen long and flattened (Fig. 1D), ground-color black, with thin gray pollinosity; syntergite 1+2 and tergites 3–5 each with one dark median triangular vitta (Fig. 1D); sternite 1 with 4 long lateral marginal setae; for morphology of sternites 5 and 9 and terminalia, see Fig. 1B, C, E, F.

FEMALE. Unknown.

##### Remarks.

Feng and Xue (2006) placed *Fannia labidocerca* into the *Fannia serena*-subgroup of the *Fannia serena*-group while recording the species from the Mt. Emei Region, Sichuan, China. However, this species should be placed into the *Fannia postica*-subgroup by sharing the following morphological characters with all other species of the subgroup: mid first tarsomere with a distinct basal tooth-like spine on ventral surface; lower calypter short, tongue-like (Fig. 1E, F).

##### Type specimens examined.

Holotype male: China, Sichuan, Emeishan, Mt. Emei, 29.59°N, 103.30°E, 3099 m, 22.VI.1984, Coll. Y. Feng (IESNU). Paratypes: 1 male, China, Sichuan, Yaan, Hanyuan, Mt. Jiaoding, 3550 m, 8.VII.1987, Coll. Y. Feng (IESNU); 1 male, China, Yunnan, Lushui, Pianma, Mt. Gaoligong, 2400 m, 24.VI.2010, Coll. Y.Y. Zhou (IESNU).

##### Distribution.

Oriental: China (Yunnan); Palearctic: China (Shaanxi, Sichuan).

#### Fannia ningxiaensis

Taxon classificationAnimaliaDipteraFanniidae

Wang & Zhang sp. n.

http://zoobank.org/C3089E00-6329-40D5-B852-ECBF3D1F70BB

Fig. 2

##### Description.

**Figure 2.**
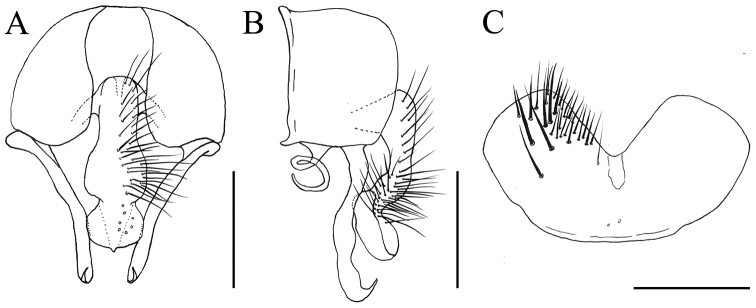
*Fannia ningxiaensis* Wang & Zhang, sp. n., male, holotype: (specimen from Ningxia, deposited in IESNU). **A.** Terminalia, ventral view **B** Terminalia, lateral view **C** Sternite 5, ventral view. Scale for **A−C** = 0.25 mm.

MALE. Body length 4.0 mm. Eye bare; postocular setae in one row, slender and curved anteriorly, occipital setae situated behind the postocular setae on vertex and in one row; fronto-orbital plate and parafacial with grayish-silvery pollinosity; frons at narrowest point slightly narrower than the distance between outer margins of posterior ocelli, about 2/3 as wide as postpedicel; frontal vitta black, linear at narrowest point; frontal setae 7 to 9, stout, nearly reaching ocellar triangle, the gaps between them without setulae; orbital setae absent; parafacial bare and narrow, at middle about 2/5 of width of postpedicel; antenna black, postpedicel about 1.5x as long as wide, arista black and ciliated, slightly swollen in basal part, the longest individual hairs shorter than aristal base; epistoma not projecting beyond vibrissal angle, vibrissal angle behind frontal angle in profile; subvibrissal setulae in one row, lateral of it with 2 or 3 fine setae; gena and genal dilation with black setulae, upper margin of gena without upcurved setae; prementum with thin grayish pollinosity, slightly shining, about 2.3x as long as wide; palpus black, claviform, about as long as prementum. Thorax ground-color black, notum with thin dark brown pollinosity, without a distinct vitta; presutural *acr* biserial, slightly stout, prescutellar pairs stout, the distance between the 2 rows of *acr* narrower than the distance between rows of *acr* and *dc*; *dc* 2+3, *ia* 0+2, *pra* 2, the anterior one about 3/5 as long as posterior notopleural seta; notopleuron bare; proepisternal setae 2, proepimeral seta 1, with about 10 slender setulae around it; basisternum, proepisternum, anepimeron, meron and katepimeron bare; katepisternal setae 1+1, katepisternum without a ventral spine, with only some fine and curved setae; anterior spiracle brown, posterior spiracle dark brown; calypters brownish with yellow-brownish margin, the lower calypter slightly smaller than the upper one and not projecting beyond the upper one. Wing brownish; veins brown; wing-base of similar color to other parts of wing; tegula black; basicosta brown; costal spine inconspicuous; node of Rs bare on ventral and dorsal surfaces; vein R_4+5_ straight, parallel to vein M_1+2_ distally; crossveins not distinctly clouded; haltere brownish-yellow. Legs entirely black; fore coxa without a spine on anterior ventral surface; fore femur with a complete row of *pv*; fore tibia without *ad* and median *p*, and with only one stout preapical *d*; fore first tarsomere with several longish basal setae on ventral surface; mid coxa without a hook-like spine or spine-like seta; mid femur with 6 to 8 stout *av* in basal part, becoming gradually shorter and denser towards apex, with a gap in preapical part, 2 to 4 comb-like setae in distal part, and with a row of stout *pv*, slightly biserial in median part, with a gap in preapical part, with 4 or 5 comb-like setae in distal part, and with a row of slender *p*; mid tibia slightly swollen in distal half, with one *ad* and one *pd* in distal half, and with numerous slender setulae on ventral surface, the longest one about 3/4 of mid tibial width in distal part; mid first tarsomere without a basal tooth-like spine on ventral surface, and with only short basal clustered setulae; hind coxa bare on posterior surface; hind femur with only one stout *av* in preapical part, with 8 to 10 stout *pv* in distal half; hind tibia with one *av*, one *ad*, and one median *d*, and with 8 or 9 slightly erect median setae on posterior surface. Abdomen oval and flattened, ground-color black, with dense grayish-blue pollinosity; syntergite 1+2 and tergites 3–4 each with one dark broad median triangular vitta, tergite 5 with one dark median stripe in basal part; sternite 1 with setulae, sternite 5 broad (Fig. 2C); cercal plate longish, from ventral view, apex of cercal plate projecting, large and rounded, as broad as middle part of cercal plate and slightly broader than the basal part (Fig. 2A); bacilliform process twisted (Fig. 2B); surstylus slender, hook-like at apex and pointed posteriorly (Fig. 2A, B).

FEMALE. Unknown.

##### Remarks.

The new species is attributed to the *spathiophora*-subgroup of the *postica*-group. It can be distinguished from a similar European species, *Fannia stigi* Rognes, 1982, by the following character states: mid first tarsomere with only short basal clustered setulae on ventral surface; hind femur with 8 to 10 stout *pv* in distal half; abdominal syntergite 1+2 and tergites 3–4 each with one dark broad median triangular vitta; apex of cercal plate projecting, large and rounded in ventral view (Fig. 2A); bacilliform process twisted (Fig. 2B), while *Fannia stigi* Rognes mid first tarsomere with a basal tooth-like spine; hind femur with 4 or 5 stout *pv* in distal half; abdominal syntergite 1+2 and tergites 3–4 each with a dark median stripe; apex of ceral plate not projecting, half round in ventral view; bacilliform process not twisted.

##### Etymology.

The specific name is derived from name of the type locality, Ningxia.

##### Types material.

Holotype male: China, Ningxia, Guyuan, Jingyuan, Dongshanpo, 2200 m, 27.VI.2008, Coll. M.F. Wang (IESNU). Paratype: 1 male, China, Ningxia, Guyuan, Jingyuan, Dongshanpo, 2000 m, 27.VI.2008, Coll. M.F. Wang (IESNU).

##### Distribution.

Palearctic: China (Ningxia).

#### Fannia nudifemorata

Taxon classificationAnimaliaDipteraFanniidae

Wang & Zhang, 2011

Fannia nudifemorata Wang & Zhang, 2011: 12.

##### Type specimens examined.

Holotype male: China, Yunnan, Yulongxueshan, 27.09°N, 100.25°E, 3200 m, 24.V.2007, Coll. W.X. Dong (IESNU). Paratype: 1 male, same locality and time, Coll. S.C. Bai (IESNU).

##### Distribution.

Oriental: China (Yunnan).

#### Fannia postica

Taxon classificationAnimaliaDipteraFanniidae

(Stein, 1895)

Fannia postica Stein, 1895: 89.
Fannia postica : Hennig 1955: 24; Chillcott 1961: 103; Pont 1986: 53; Rozkošný et al. 1997: 27; Xue and Wang 1998: 819; Wang and Xue 2002: 57; Wang et al. 2006: 555.

##### Material examined.

China: Heilongjiang: 2 male, Xilinji, 53.48°N, 122.37°E, 19.VI.1986, Coll. C.Y. Cui (IESNU).

##### Distribution.

Nearctic: throughout North America; Palearctic: Austria, Belgium, Bulgaria, China (Heilongjiang), Czech Republic, Denmark, Finland, former Yugoslavia, France, Germany, Iceland, Ireland, Italy, Luxembourg, Norway, Poland, Romania, Slovakia, Spain, Sweden, Switzerland, United Kingdom (England).

#### Fannia ringdahlana

Taxon classificationAnimaliaDipteraFanniidae

Collin, 1939

Fannia ringdahlana Collin, 1939: 143.
Fannia ringdahlana : Hennig 1955: 20; Pont 1977: 449; Pont 1986: 54; Fan 1992: 216; Wang and Wu 1996: 66; Rozkošný et al. 1997: 22; Xue and Wang 1998: 815; Wang and Xue 2002: 57; Wu and Wang 2002: 563; Wang et al. 2004: 34; Wang et al. 2006: 555.

##### Material examined.

China: Jilin: 2 male, Mt. Changbai, Xiaotianchi, 42.58°N, 128.30°E, 25.VII.1982, Coll. L.Y. Gao (IESNU); 2 male, Mt. Changbai, 42.33°N, 127.27°E, 18.VII.1988, [collector unknown]. Shanxi: 1 male, Ningwu, Mt. Luya, 38.73°N, 111.93°E, 12.VI.1987, Coll. M.F. Wang (IESNU). Sichuan: 2 male, Jiuzaigou, 33.26°N, 103.91°E, 2800 m, 1.VI.2006, Coll. Y. Zhu (IESNU); 3 male, same locality, 2.VI.2006, Coll. D. Jing (IESNU); 9 male, same locality, 3.VI.2006, Coll. D. Wang (IESNU); 1 male, Daocheng, Kasi, 29.04°N, 100.31°E, 2750–3000 m, 12.VII.2006, Coll. C.T. Zhang (IESNU). Yunnan: 1 male, Deqin, Mt. Meili, 28.49°N, 98.93°E, 4000–4200 m, 2.VII.2006, Coll. Y. Wang (IESNU); 1 male, Xianggelila, Bitahai, 27.80°N, 99.90°E, 3700 m, 2.VII.2006, Coll. B.F. Wang (IESNU); 5 male, same locality and time, Coll. L. Chang (IESNU); 5 male, same locality and time, Coll. M.F. Wang (IESNU).

##### Distribution.

Oriental: China (Taiwan, Yunnan); Palearctic: China (Jilin, Shanxi, Sichuan), Japan, Sweden, United Kingdom.

#### Fannia spathiophora

Taxon classificationAnimaliaDipteraFanniidae

Malloch, 1918

Fannia spathiophora Malloch, 1918: 294.
Fannia spathiophora : Chillcott 1961: 112; Wang and Wu 1996: 66; Rozkošný et al. 1997: 23; Xue and Wang 1998: 815; Wang and Xue 2002: 57; Wu and Wang 2002: 563; Wang et al. 2004: 34; Wang et al. 2006: 556.

##### Material examined.

China: Heilongjiang: 1 male, Wuying, 48.11°N, 129.24°E, 16.VII.1977, Coll. C.Y. Cui (IESNU); 1 male, Guyuan, 50.58°N, 123.70°E, 26.VI.1980, Coll. C.Y. Cui (IESNU); 1 male, Bizhou, 51.94°N, 124.60°E, 13.VII.1980 [collector unknown] (IESNU). Jilin: 1 male, Baihe, 42.58°N, 128.04°E, 20.VI.1980, Coll. Z.Y. Ma (IESNU); 1 male, Mt. Changbai, 42.33°N, 127.27°E, 19.VII.1986 [collector unknown] (IESNU); 1 male, Mt. Changbai, 42.33°N, 127.27°E, 15.VII.1990 [collector unknown] (IESNU). Liaoning: 2 male, Xinbin, Gangshan, 41.72°N, 125.02°E, -.VI.1981, Coll. Z.Y. Ma (IESNU); 1 male, same locality, 08.IX.1990, [collector unknown] (IESNU); 2 male, Benxi, Yanghugou, 41.30°N, 123.73°E, 01.VII.1993, Coll. Y.S. Cui (IESNU); 1 male, same locality, 01.VII.1993, Coll. C.T. Zhang (IESNU); 1 male, Huanren, 41.27°N, 125.35°E, 09.VI.1994, Coll. D. Wei (IESNU); 3 male, Qianshan, 41.03°N, 123.13°E, 25.VI.2007, Coll. M.F. Wang (IESNU). Shanxi: 1 male, Hunyuan, 39.70°N, 113.68°E, 12.VII.1985, Coll. M.F. Wang (IESNU).

##### Distribution.

Nearctic: Canada (Labrador, Northwest Territories, Ontario), USA (Alaska, south to Arizona & New Mexico, Minnesota); Palearctic: China (Hebei, Heilongjiang, Jilin, Liaoning, Shanxi), throughout Europe, Japan.

#### Fannia slovaca

Taxon classificationAnimaliaDipteraFanniidae

Gregor & Rozkošný, 2005

Fannia slovaca Gregor & Rozkošný, 2005: 519.

##### Distribution.

Palearctic: Slovakia.

#### Fannia stigi

Taxon classificationAnimaliaDipteraFanniidae

Rognes, 1982

Fannia stigi Rognes, 1982: 325.
Fannia stigi : Wang, Li and Zhang 2011: 15.
Fannia tigripeda : Xue, Wang and Li 2001: 225–226; Wang and Xue 2002: 57; Su and Wang 2004: 112.

##### Material examined.

China: Jilin: Mt. Changbai, 42.33°N, 127.27°E, 1700 m, 28.VI.1997, Coll. W.Q. Xue (IESNU). Shanxi: 1 male, Ningwu, 38.73°N, 111.93°E, 07.VI.1982, Coll. M.F. Wang (IESNU).

##### Distribution.

Palearctic: China (Jilin, Shanxi), Norway, Sweden.

#### Fannia subaethiops

Taxon classificationAnimaliaDipteraFanniidae

Wang & Zhu sp. n.

http://zoobank.org/A9405B63-59AD-4D73-829F-F0364A86A69A

Fig. 3

##### Description.

**Figure 3.**
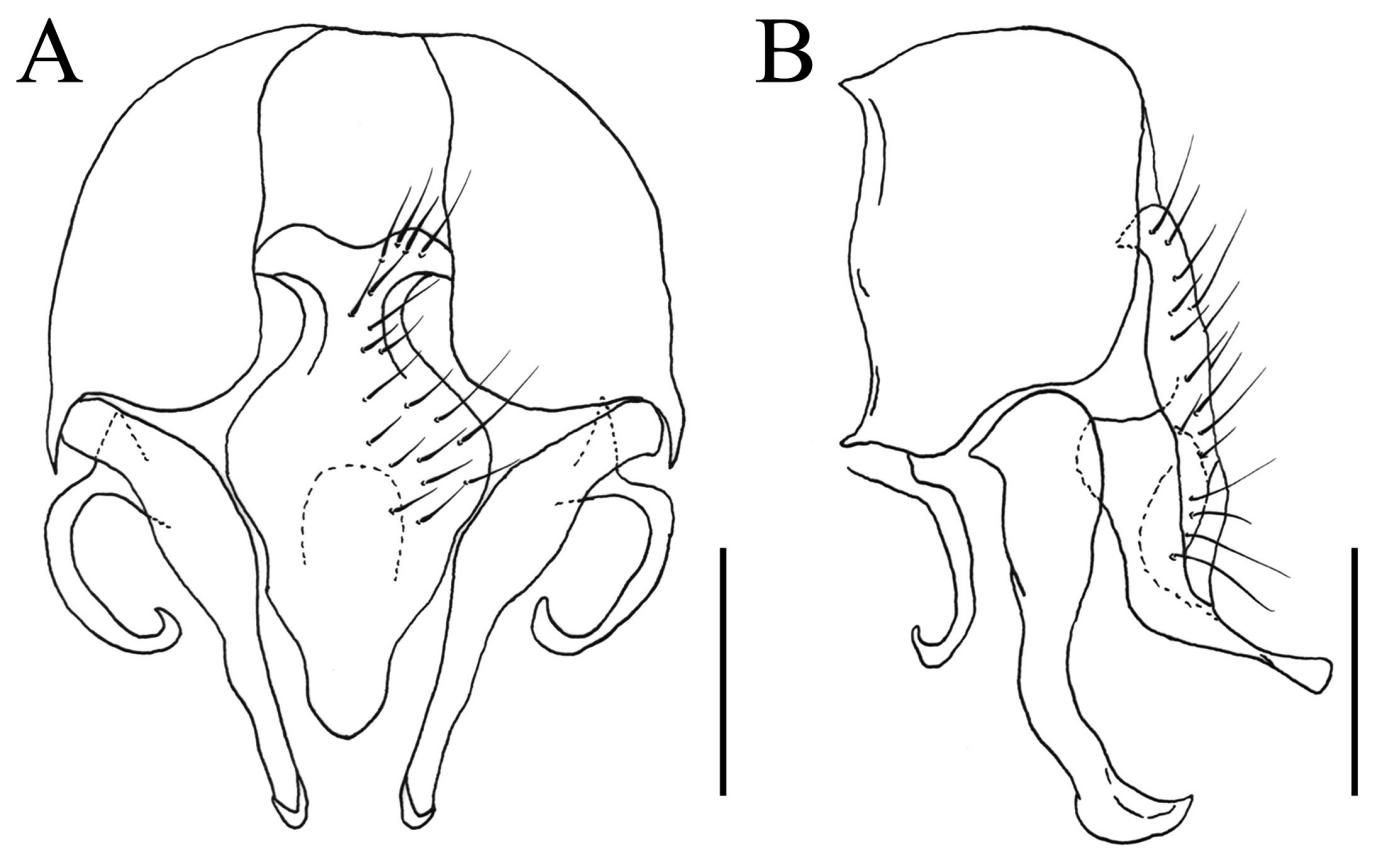
*Fannia subaethiops* Wang & Zhu, sp. n., male, holotype: (specimen from Heilongjiang, deposited in SHEM). **A** Terminalia, ventral view **B** Terminalia, lateral view. Scale for **A−B** = 0.25 mm.

MALE. Body length 5.0 mm. Eye bare; upper inner facets larger than the remaining facets; postocular setae in one row, short and neatly arranged, occipital setae absent; fronto-orbital plate and parafacial with grayish-silvery pollinosity; frons at narrowest point slightly wider than the distance between outer margins of posterior ocelli, about as wide as postpedicel; frontal vitta black, with grayish-silvery pollinosity, at narrowest point about as wide as fronto-orbital plate; frontal setae 5, stout, nearly reaching ocellar triangle, the gaps between them without setulae, orbital setae absent; parafacial bare, at middle about 1/2 as wide as postpedicel; antenna black, postpedicel about 1.5x as long as wide, arista ciliated, slightly swollen in basal part, the longest individual hairs shorter than aristal base; epistoma not projecting beyond vibrissal angle, vibrissal angle behind frontal angle in profile; subvibrissal setulae in one row, lateral of it with several setae; gena and genal dilation with black setulae, upper margin of gena without upcurved setae; prementum shining, without distinct pollinosity, about 2.0x as long as wide; palpus black, claviform, slightly longer than prementum. Thorax ground-color black, notum with dark brown pollinosity, without a distinct vitta; presutural *acr* biserial, slightly stout, only prescutellar pairs stout, the distance between 2 rows of *acr* narrower than the distance between rows of *acr* and *dc*; *dc* 2+3, *ia* 0+2, *pra* 2, the anterior one stout, about 2/3 as the length of posterior notopleural seta; notopleuron bare; proepisternal setae 2, proepimeral seta 1, lower part of proepimeral seta with one short setula; basisternum, proepisternum, anepimeron, meron and katepimeron bare; katepisternal setae 1+1, katepisternum without a ventral spine; spiracles brown; calypters brownish-yellow, the lower one slightly projecting beyond the upper one. Wing brownish; veins dark brown; wing-base of same color as other parts of wing; tegula dark brown; basicosta brownish-yellow; costal spine conspicuous, about 2/3 of the length of crossvein r-m; node of Rs bare on ventral and dorsal surfaces; vein R_4+5_ straight, veins M_1+2_ and R_4+5_ converging distally; crossveins not clouded; haltere brown. Legs entirely black, except knees yellow; fore coxa without a anterior spine on ventral surface; fore femur with a stout row of *pv*; fore tibia without *ad* and median *p*, with only one *d* and one *v* in apical part; fore first tarsomere with few longish basal setae on ventral surface; mid coxa without a hook-like spine or spine-like seta; mid femur with a row of stout and sparse *av* in basal half, becoming shorter and denser towards apex, with a gap in preapical part, 2 or 3 comb-like setae in distal part, a complete row of stout *pv*, slightly biserial in median part, and a row of slender *p*; mid tibia slightly narrowing in basal half, gradually swollen towards apex, about 2.0x as wide in distal part as wide in basal part, with one *ad* and one *pd* in distal half, and with numerous slender setulae on ventral surface, the longest one about 3/4 as long as mid tibial width in distal part; mid first tarsomere without a basal tooth-like spine on ventral surface, with only short basal clustered setulae; hind coxa bare on posterior surface; hind femur with only one stout *av* and 3 or 4 *pv* in preapical part; hind tibia with one *av*, one *ad* and one *d*. Abdomen long and flattened, ground-color black, with grayish-brown pollinosity; syntergite 1+2 and tergites 3–4 each with an inverted T-shaped dark mark, each tergite with stout lateral marginal setae; sternite 1 broad, with 4 long setae on each lateral margin, sternites 2 to 4 narrow, with long setulae, sternite 5 with slightly dense setae in posterior margin; cercal plate longish, from ventral view, cercal plate slightly indented in each lateral margin, middle part of cercal plate strongly broader than the apex and the basal part (Fig. 3A); bacilliform process curved (Fig. 3A, B); surstylus slender, curved at apex and pointed posteriorly (Fig. 3A, B).

FEMALE. Unknown.

##### Remarks.

The new taxon is similar to the holarctic species *Fannia aethiops* Malloch, 1913 but differs from it for the following character states: frontal setae only 5; anterior *pra* about 2/3 as long as posterior notopleural seta; sternite 1 with 4 long setae on each lateral margin; in ventral view, cercal plate broadest in median part, slightly indented in each lateral margin (Fig. 3A); bacilliform process curved (Fig. 3A & B), while *Fannia aethiops* Malloch frontal setae 9; anterior *pra* about 1/2 as long as posterior notopleural seta; sternite 1 with 1–2 long setae on each lateral margin; in ventral view, cercal plate not indented in each lateral margin; bacilliform process not curved.

##### Etymology.

This specific name refers to the similarity between the new species and *Fannia aethiops* Malloch.

##### Types material.

Holotype male: China, Heilongjiang, Yichun, Wuying, 3.V.1975, Coll. S.Y. Fang (SHEM).

##### Distribution.

Palearctic: China (Heilongjiang).

#### Fannia umbratica

Taxon classificationAnimaliaDipteraFanniidae

Collin, 1939

Fannia umbratica Collin, 1939: 144.
Fannia umbratica : Hennig 1955: 90; Pont 1986: 57; Rozkošný et al. 1997: 47.

##### Distribution.

Palearctic: throughout Europe.

#### Fannia umbrosa

Taxon classificationAnimaliaDipteraFanniidae

(Stein, 1895)

Fannia umbrosa (Stein, 1895): 75.
Fannia umbrosa : Hennig 1955: 90; Pont 1986: 57; Rozkošný et al. 1997: 47.

##### Distribution.

Palearctic: throughout Europe.

## Supplementary Material

XML Treatment for Fannia

XML Treatment for Fannia aethiops

XML Treatment for Fannia ardua

XML Treatment for Fannia bigelowi

XML Treatment for Fannia coculea

XML Treatment for Fannia discoculea

XML Treatment for Fannia gotlandica

XML Treatment for Fannia labidocerca

XML Treatment for Fannia ningxiaensis

XML Treatment for Fannia nudifemorata

XML Treatment for Fannia postica

XML Treatment for Fannia ringdahlana

XML Treatment for Fannia spathiophora

XML Treatment for Fannia slovaca

XML Treatment for Fannia stigi

XML Treatment for Fannia subaethiops

XML Treatment for Fannia umbratica

XML Treatment for Fannia umbrosa
